# Promising antimicrobials from *Phoma* spp.: progress and prospects

**DOI:** 10.1186/s13568-022-01404-y

**Published:** 2022-05-23

**Authors:** Mahendra Rai, Beata Zimowska, Aniket Gade, Pramod Ingle

**Affiliations:** 1 Department of Biotechnology, Sant Gadge Baba Amravati University, Amravati, 444 602 Maharashtra India; 2 Department of Microbiology, Nicolaus Copernicus University, 87-100 Toruń, Poland; 3 Department of Plant Protection, Institute of Plant Pathology and Mycology, University of Life Sciences in Lublin, 7 K. St. Leszczyńskiego Street, 20-069 Lublin, Poland

**Keywords:** *Phoma* spp., Multidrug-resistance, Antibiotics, Bioactive metabolites, Silver nanoparticles

## Abstract

The increasing multidrug-resistance in pathogenic microbes and the emergence of new microbial pathogens like coronaviruses have necessitated the discovery of new antimicrobials to treat these pathogens. The use of antibiotics began after the discovery of penicillin by Alexander Fleming from *Penicillium chrysogenum*. This has attracted the scientific community to delve deep into the antimicrobial capabilities of various fungi in general and *Phoma* spp. in particular. *Phoma* spp. such as *Phoma arachidicola, P. sorghina, P. exigua* var. *exigua*, *P. herbarum, P. multirostrata, P. betae, P. fimeti, P. tropica,* among others are known to produce different bioactive metabolites including polyketides, macrosporin, terpenes and terpenoids, thiodiketopiperazines, cytochalasin derivatives, phenolic compounds, and alkaloids. These bioactive metabolites have already demonstrated their antimicrobial potential (antibacterial, antifungal, and antiviral) against various pathogens. In the present review, we have discussed the antimicrobial potential of secondary metabolites produced by different *Phoma* species. We have also deliberated the biogenic synthesis of eco-friendly antimicrobial silver nanoparticles from *Phoma* and their role as potential antimicrobial agents.

## Key points

- Growing multidrug-resistance and emerging pathogens need new antimicrobial drugs
- Different species of *Phoma* produce antimicrobial metabolites
- *Phoma* spp. are potential synthesizers of silver nanoparticles demonstrating antimicrobial activity.

## Introduction

There are terrifying global reports of the multidrug-resistance in pathogens that are not responding to the available antibiotics (Wencewicz 2019). The main reasons for developing resistance by microbes include misuse and overuse of antibiotics, and environmental factors (Ghosh et al. 2020; Christaki et al. 2020). This problem of antibiotic resistance has garnered the attention of the scientific community, policymakers, and the public at large from all over the world, and it is a global health challenge (Markley and Wencewicz 2018; Hu et al. 2021).

The new and emerging diseases caused by microbes are major threat to mankind. The recent emergence of the COVID-19 pandemic caused by SARS-CoV-2 is a burning example that has devastated human life globally. The current burden of co-infections and superinfections such as mucormycosis in COVID-19 patients is also a great issue that emphasizes the discovery of new antimicrobials (Feldman and Anderson 2021). Moreover, there has been huge concern about re-emerging microbial diseases such as malaria, tuberculosis, influenza, cholera, pertussis, etc.

Unfortunately, for more than three decades, no new antibiotics have been discovered (Böttcher et al. 2021), and therefore, these facts warrant the discovery of new antibiotics and/ or search for new alternatives from natural products such as plants and microbes to tackle such a grave problem (WHO Newsletter 2020). Among the microbes, fungi play a key role in the production of antimicrobials. The serendipitous discovery of penicillin by Alexander Fleming (1929) from *Penicillium notatum* and *P. chrysogenum* is the best example (Zhu et al. 2014). Other potential antibiotics produced by fungi include cephalosporins and griseofulvin. Several species of *Phoma* such as *P. arachidicola, P. sorghina, P. exigua* var. *exigua*, *P. herbarum, P. multirostrata, P. betae,* and *P. fimeti* are pigment-producing (Chande et al. 1899) and some *Phoma* species have already demonstrated the antimicrobial potential against various fungi (Aoyagi et al. 2007; Hussain et al. 2014), bacteria (Huang et al. 2017; Chen et al. 2019) and viruses (Liu et al. 2019; Peng et al. 2020). They produce secondary metabolites with antimicrobial potential. These bioactive compounds include polyketides like anthraquinones and diphenyl ether derivatives; ergocytochalasin A, macrosporin, thiodiketopiperazines, cytochalasin derivatives, and alkaloids. The antimicrobial metabolites producing species of *Phoma* can be harnessed to treat various microbial pathogens.

The present review is focused on the antimicrobial potential of secondary metabolites produced by different terrestrial, marine or endophytic *Phoma* species. Moreover, the biogenic synthesis of eco-friendly antimicrobial silver nanoparticles produced from *Phoma* and their role as potential antimicrobial agents have been discussed. The review is timely as so far there is no review available on the antimicrobial nature of metabolites produced by different *Phoma s*pecies.

## *Phoma:* the producer of novel bioactive metabolites

**Fig. 1:**
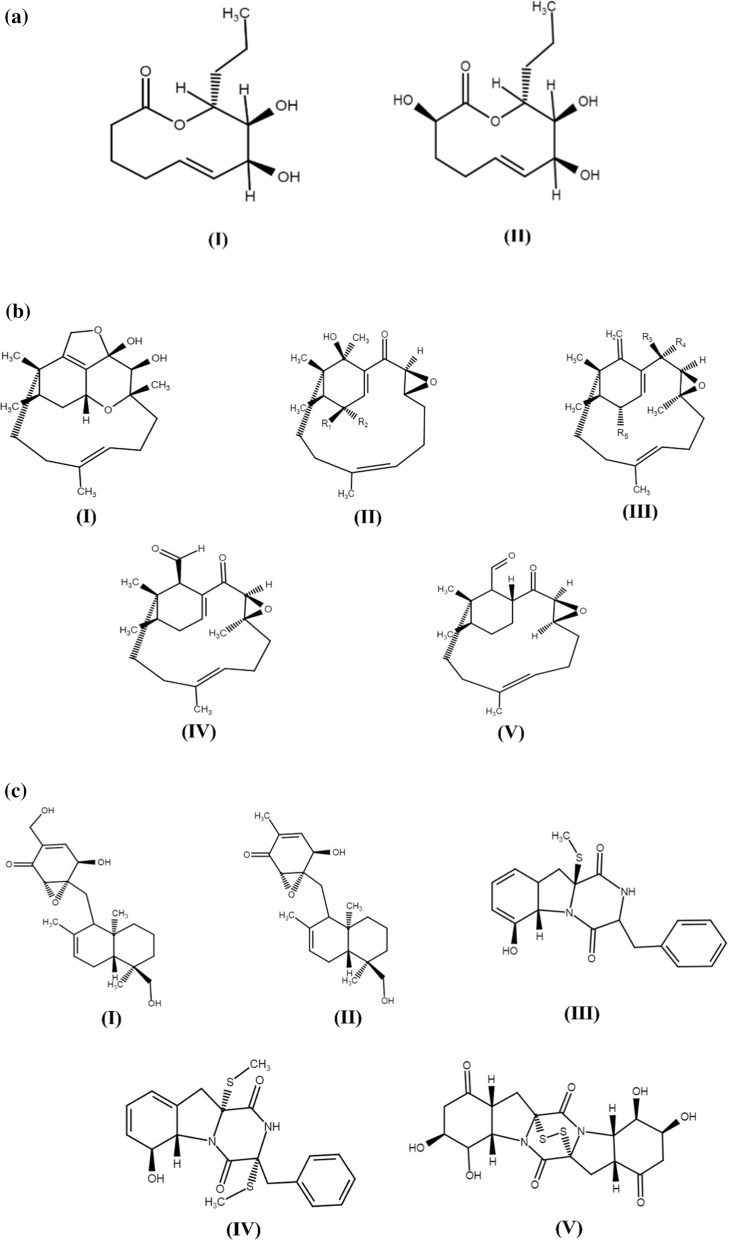
**a** Chemical structures of bioactive compounds recovered from *P. herbarum*. (I) Herbarumin I and (II) Herbarumin II. **b** Chemical structures of bioactive metabolites isolated from marine *Phoma* species. (I) Phomactin A. (II) Phomactin B (R1 = H; R2 = OH) & B1 (R1 = OH; R2 = H). (III) Phomactin B2 (R3 & R4 = O; R5 = OH). (IV) Phomactin C. (V) Phomactin D. **c** Chemical structures of bioactive metabolites obtained from marine *Phoma* sp. (I) Epoxyphomalin A. (II) Epoxyphomalin B; and from *Phoma* sp. OUCMDZ-1847. (III) Phomazine A. (IV) Phomazine B. (V) Phomazine C (Rai et al. 2018) reprinted with permission

**Fig. 2:**
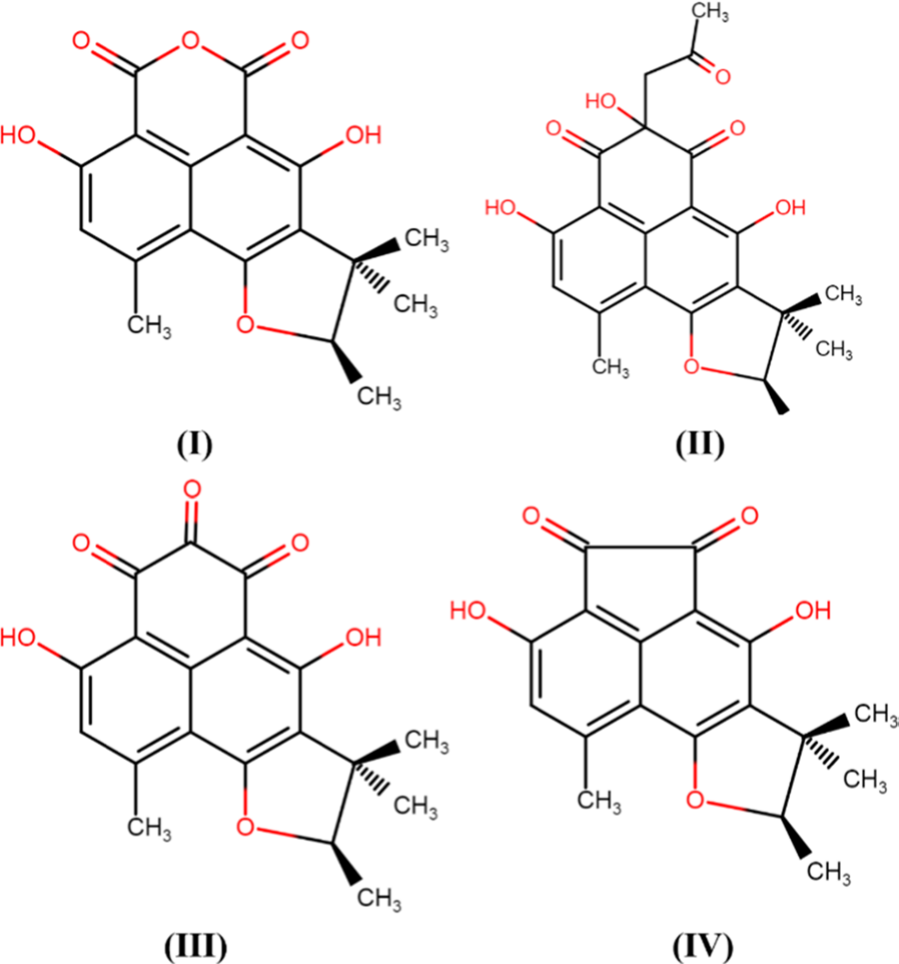
Phytochemicals identified from an endophytic *Phoma* sp. (I) Sclerodin, (II) 8,9-dihydro-3,5,7-trihydroxy-1,8,8,9-tetramethyl-5-(2-oxopropyl)-4H-phenaleno[1,2-b]furan-4,6(5H)-dione, (III) Atrovenetinone, and (IV) Sclerodione. Reprinted from Hussain et al. (2015) under Creative Common Rights Licence

The *Phoma* spp. are widely distributed as pathogens of plants, animals, and humans, and also in soil, water and air (Rai 2002). The *Phoma* spp. secrete various metabolites that have already demonstrated antimicrobial potential (Rai et al. 2009a, b, c, 2018; Herath et al. 2009). Not only terrestrial but marine and endophytic species of *Phoma* are also responsible for the production of antimicrobial metabolites (Hoffman et al. 2008; Bhimba et al. 2012; Elsebai et al. 2016, 2018). A large number of metabolites with unique structures, and potential biological and pharmacological activities have been reported from the marine *Phoma* species particularly *P. sorghina*, *P. herbarum,* and *P. tropica*. These metabolites generally include lactones, quinine, diterpenes, phthalate, enolides, and anthraquinones (Fig. 1a–c). which have shown a broad range of bioactivities including antimicrobial, anticancer, radical scavenging, and cytotoxic (Rai et al. 2018, 2020). There are several reports which provide conclusive evidence that endophytic *Phoma* species living in plants secrete potential antimicrobial compounds (Fig. 2) (Hussain et al. 2015; Huang et al. 2017; da Silva et al. 2017; de Vries et al. 2018; Nalli et al. 2019; El-Zawawy et al. 2020; Li et al. 2020; Rai et al. 2020; Hu et al. 2021). For example, the compounds like α-tetralone derivative (3S)-3,6,7-trihydroxy-α-tetralone, together with cercosporamide, β-sitosterol, and trichodermin reported from the ethyl acetate extract of endophytic *Phoma* sp. (ZJWCF006) isolated from *Arisaema erubescens* (Wang et al., 2012)*.* These compounds were found to be effective against the plant pathogenic fungi such as *Fusarium oxysporum*, *Rhizoctonia solani*, *Colletotrichum gloeosporioides*, *Magnaporthe oryzae,* and plant pathogenic bacteria including *Xanthomonas campestris* and *X. oryzae*.

**Fig. 3:**
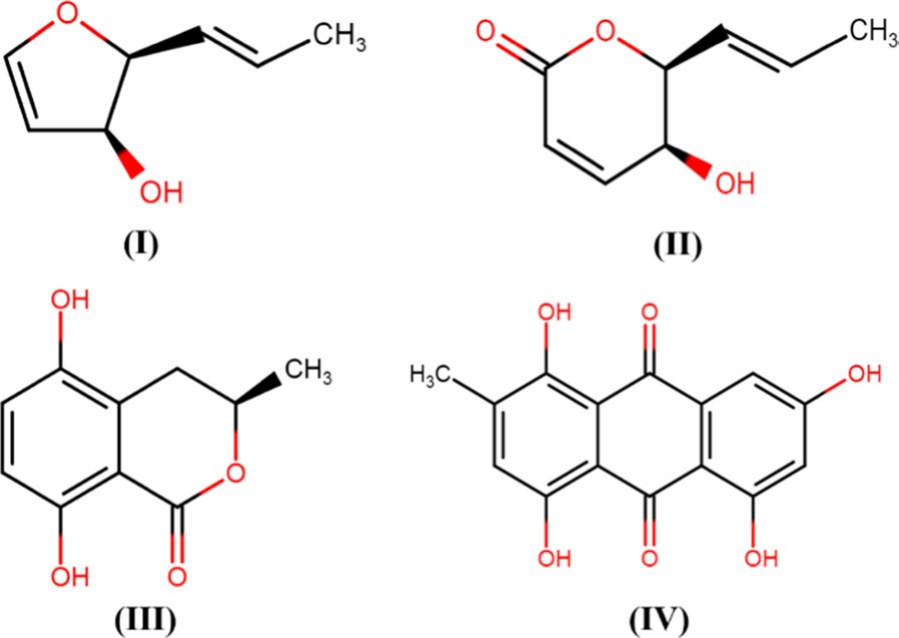
Structures of compounds isolated from *Phoma* sp. (I) phomafuranol, (II) phomalacton, (III) (3R)-5-hydroxymellein, and (IV) emodin (Hussain et al. 2014)—redrawn using free access MedChem Designer 5.5

Many species of *Phoma* have demonstrated remarkable antimicrobial activities. For example, Hussain et al. (2014) isolated phomafuranol (I), phomalacton (II), (3R)-5-hydroxymellein (III), and emodin (IV) (Fig. 3) from the ethyl acetate fractions of *Phoma* spp. recovered from *Fucus serratus.* which demonstrated potential inhibitory activities including antibacterial, antifungal, and antialgal.

**Fig. 4:**
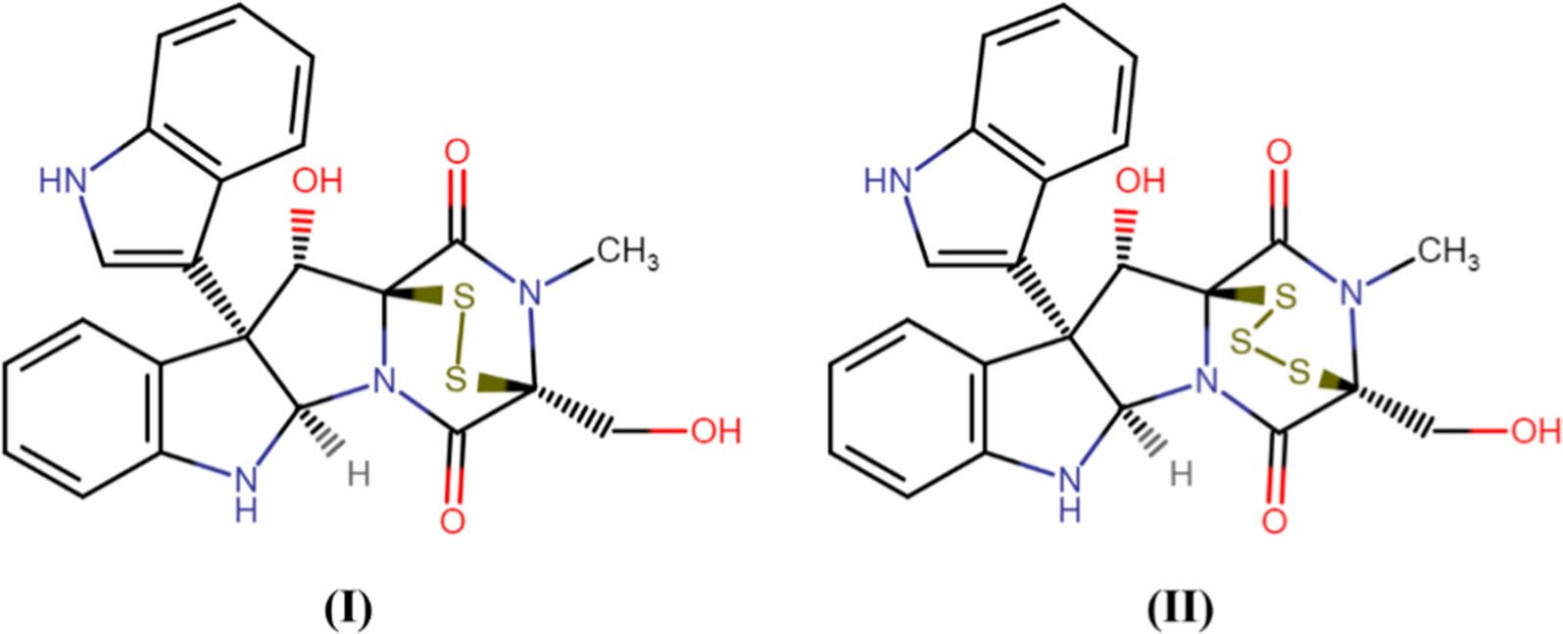
Thiodiketopiperazine derivatives, Compound I and II, from *Phoma* sp. (Arora et al. (2016); Redrawn using free access MedChem Designer 5.5

Arora et al. (2016) screened endophytes isolated from *Glycyrrhiza glabra* and reported the presence of *Phoma* spp. which was closely related to *P. cucurbitacearum*. Further, the authors isolated two thiodiketopiperazine derivatives (Fig. 4) from the extract of this species of *Phoma* which showed remarkable antibacterial activity against *Staphylococcus aureus* and *S. pyogene* Moreover, these compounds significantly inhibited the biofilm formation ability of both the pathogens singly and in combination with ciprofloxacin and ampicillin in a synergistic way. Endophytic *Phoma* spp. (URM 7221) isolated from the leaves of *Schinus terebinthifolius* effectively inhibited *S. aureus*, MRSA, *B. subtilis,* and *E. faecalis* (de Silva et al., 2017). The potential of *Phoma* sp. was attributed to the production of phenolic compounds and steroids.

In another study, Chen et al. (2019) reported that *Phoma* species SYSU-SK-7 inhabiting endophytically in mangrove plant *Kandelia candel* contains polyketides that have shown significant activity against *Pseudomonas aeruginosa, Staphylococcus aureus* followed by *Candida albicans.* Recently, Peng and his colleagues (2020) reported ergocytochalasin A from *P. multirostrata* which was found as an endophyte in *Parasenecio albus.* The bioactive compound demonstrated strong activity against different pathogenic viruses including Human dengue virus type 3 (DV3), influenza A virus (H1N1), and respiratory syncytial virus (RSV).

## Secondary metabolites are responsible for antimicrobial activity

**Fig. 5:**
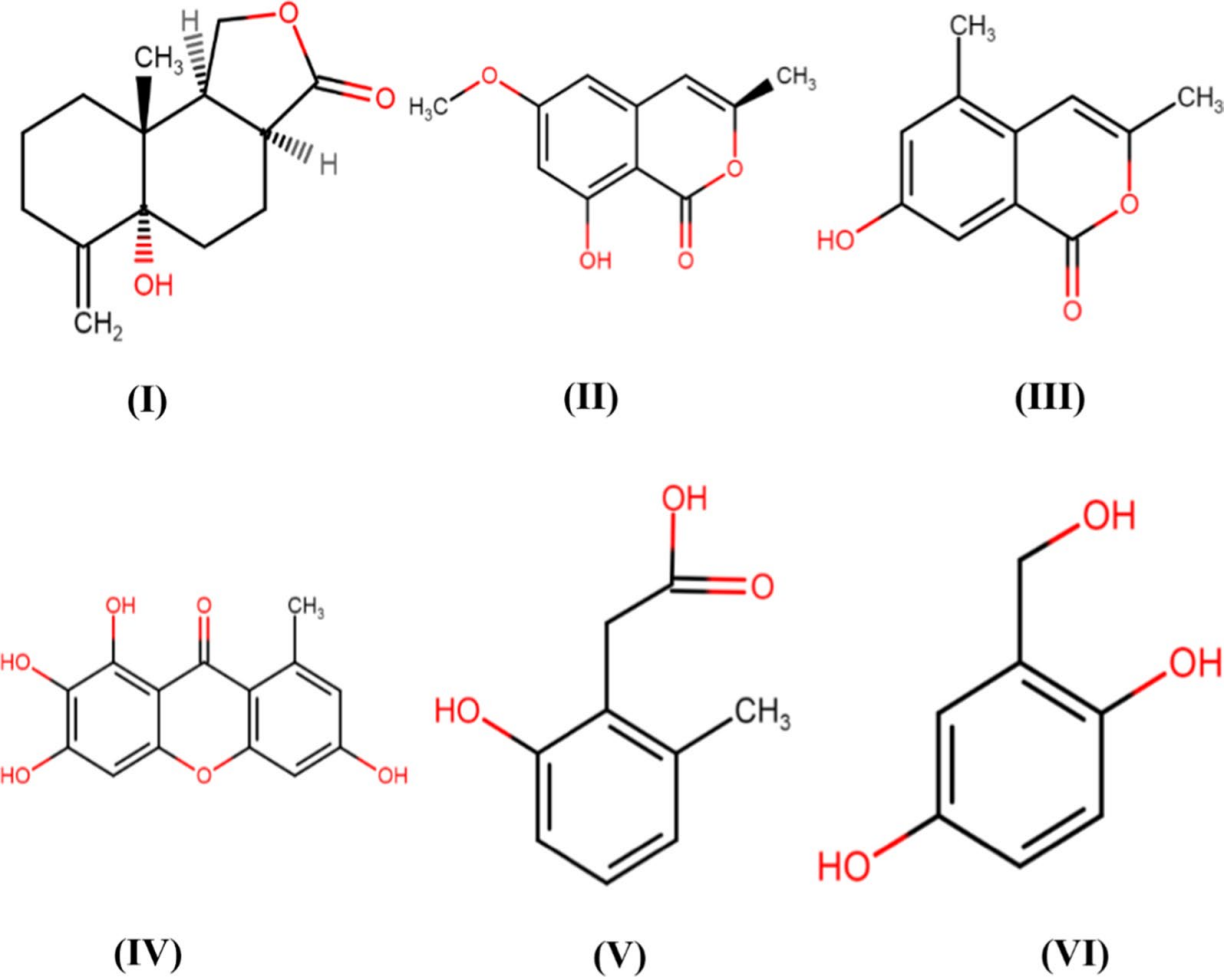
Bioactive compounds recovered from both culture of an endophytic *Phoma* sp. isolated from the roots of *Aconitum vilmorinianum*, (I) Phomanolide, (II) (–)-6-methoxymellein, (III) 7-hydroxy-3, 5-dimethyl-isochromen-1-one, (IV) Norlichexanthone, (V) 6-methylsalicylic acid, and (VI) Gentisyl alcohol (Liu et al. 2019) Redrawn using free access MedChem Designer 5.5

The secondary metabolites such as anthraquinones are secreted by *Phoma* spp. including *P. herbarum, P. exigua* var. *exigua, P. sorghina, P. macrostoma, P. glomerata, P. macdonaldii, P. tracheiphila, P. multirostrata, P. proboscis,* and *P. foveata*, etc. (Rai et al. 2009a, b, c, 2021a, b). As shown in Fig. 5, the different bioactive secondary metabolites reported from *Phoma* spp include. α-Pyrone derivatives (Sang et al. 2017), isocoumatins (Hussain et al. 2014; Shi et al. 2017); anthraquinones and xanthones (Xia et al. 2015; Liu et al. 2019); thiodiketopiperazines, phomazines (Arora et al. 2016); cytochalasin derivatives (Peng et al. 2020), and diphenyl ether derivatives (Sumilat et al. 2017), tetrasubstituted furopyrans, chenopodolans E (Evidente et al. 2016), xyloketals and chromones (Kim et al. 2018), meroterpenoids and diterpenoids (Xu et al. 2016), alkaloids such as phomapyrrolidones (Wijeratne et al. 2013), polyketides, phomaketides (Li et al. 2020) produced by different *Phoma* spp. A detailed account of different *Phoma* spp., secondary bioactive compounds, and antimicrobial activities have been given in Table 1.

**Table 1:** Antimicrobial activity of *Phoma* metabolites

Species	Compound	Activity	Host plant/Source	Reference
*Phoma* sp.	Sclerodione	Antifungal: *Eurotium repens* *Ustilago violacea*		Hussain et al. (2015)
	8,9-dihydro-3,5,7-trihydroxy-1,8,8,9-tetramethyl-5-(2-oxopropyl)-4H-phena-leno[1,2-b]furan-4,6(5H)-dione	Antifungal: *Mycotypha microspora*		Hussain et al. (2015)
	Atrovenetinone	Antifungal: *Fusarium oxysporum* *E. repens* *Ustilago violacea*		Hussain et al. (2015)
	sclerodin; 8,9-dihydro-3,5,7-trihydroxy-1,8,8,9-tetramethyl-5-(2-oxopropyl)-4H-phenaleno[1,2-b]furan-4,6(5H)-dione, atrovenetinone; and sclerodione	Antibacterial: *Bacillus megaterium*		Hussain et al. (2015)
*Phoma* sp.	4-acetylpyrenophorol	Antibacterial: *E. coli, B. megaterium* Antifungal: *Microbotryum violaceum* Antialgal: *Chlorella fusca*	*Lycium* *intricatum*	Zhang et al. (2008)
	4α-acetyldihyd- ropyrenophorin			
	cis-dihydropyrenophorin			
	tetrahydropyrenophorin			
	7α-acetyl- seco-dihydropyrenophorin			
	seco-dihydropyrenophorin	Antibacterial: *E. Coli,* *B. megaterium* Antifungal: *M. violaceum*		
	seco-dihydropyrenopho- rin-1,4-lactone			
	pyrenophorin	Antifungal: *Microbotryum violaceum* Antialgal: *C. fusca*		
	4,4⬘- diacetylpyrenophorol			
*Phoma* sp. URM 7221		Antibacterial: *S. aureus,* *B. subtilis*	*Schinus terebinthifolius*	da Silva et al. (2017)
*Phoma* sp*.*	viridicatol, tenuazonic acid, alternariol, and alternariol monomethyl ether	Antifungal: *Fusarium graminearum, F* *lateritium, F. sporotrichioides, F* *avenaceum, Trichoderma longibrachiatum,* *Aspergillus flavus* and *Alternaria alternata*	*Eleusine coracana*	Mousa et al. (2015)
*Phoma* sp.	Phomodione	Antifungal: *Pythium ultimum, Sclerotinia sclerotiorum, Rhizoctonia solani*	*Saurauia scaberrinae*	Hoffman et al. (2008)
		Antibacterial: *S. aureus, E. coli*		
*Phoma herbarum* VB7	Phalate derivates	Antibacterial: *Vibrio cholerae, Micrococcus* *luteus, Salmonella thyphi, S* *aureus*	Mangrove leaves	Bhimba et al. (2012)
*Phoma* sp.	polyketide derivatives	Antibacterial: *E. coli, B. subtilis*, *Mycobacterium phlei*,*S. aureus*	*Ectyplasia perox*	Elsebai et al. (2016)
*Phoma multirostrata* PUTY3	Crude extract	Antibacterial: *Pseudomonas aeruginosa*	*Carica papaya*	Ahmed and Sarma (2020)
*Phoma medicaginis*	Crude extract	Antibacterial: *S. aureus, E. coli, P. aeruginosa*	*Mikania cordata*	Jayatilake and Munasinghe (2020)
*Phoma hedericola*		Antibacterial: *B. subtilis*, *Bacillus licheniformis*, *Micrococcus luteus, P. aeruginosa*	*Calotropis procera*	Juyal et al. (2017)
*Phoma sorghina, Phoma exigua, Phoma herbarum, Phoma fimeti*	pigments	Antibacterial:*S. aureus, P. aeruginosa, B. subtilis* and *Proteus vulgaris*		Kadu (2021)
*Phoma moricola*	(3S)-3, 6, 7-trihydroxy-α-tetralone	Antibacterial: *E. coli*, *Klebsiella pneumoniae*, *P. vulgaris*, *P. aeruginosa*, *Salmonella typhimurium*, *Staphylococcus aureus,* and *Streptococcus faecalis*	*Withania somnifera*	Roshan and Mohana (2021)
		Antifungal: *Alternaria brassicicola*, *A. geophila, Aspergillus flavus*, *A. fumigatus, A. ochraceus, A. tamarii, A. terreus, Curvularia tetramera*, *F. oxysporum*, *F. lateritium*, *F. equiseti, F. udum, verticillioides*, *Penicillium citrinum, P. expansum*		
*Phoma* sp*.*	flavipucine	Antifungal:*Phytophthora infestans*	*Salsola oppositifolia*	Loesgen et al. (2011)
*Phoma herbarum*	Ethyl acetate extract	Antibacterial: *Bacillus cereus*	*Urospermum picroides*	El-Zawawy et al. (2020)
*Phoma* sp.135	cryptophomic acid, cryptodiol, cryptotriol	Antibacterial:*E. coli, B. subtilis, Mycobacterium phlei, S. aureus*	Marine-derived	Elsebai et al. (2018)
*Phoma * sp. L28	*7-(*γ,γ)*-dimethylallyloxymacrosporin, macrosporin,* *7-methoxymacrosporin, tetrahydroaltersolanol B, altersolanol L, ampelanol*	Antifungal: *Colletotrichum musae, Colletotrichum gloeosporioides, Fusarium graminearum, Penicillium italicum, F. oxysporum.* f. sp*. lycopersici Rhizoctonia* *solani*	mangrove	Huang et al. (2017)
	*macrosporin*	Antifungal: *F. graminearum*		
*Phoma* sp. JS752	barceloneic acid C	Antibacterial: *Listeria monocytogenes, Staphylococcus pseuditermedius*	*Phragmites communis*	Xia et al. (2015)
*Phoma macrostoma*	macrooxazole C	Antibacterial: *B. subtilis* Antifungal: *Mucor hiemalis*	*Circium arvense*	Matio Kemkuignou et al. (2020)
	macrocidin A macrooxazole B, macrooxazole C, macrocidin Z	Antibacterial:*S. aureus*		
*Phoma herbarum* YG5839	tyrosine derivative, terezine derivatives	Antifungal: *F. oxysporum*, *F. graminearum*, *P. italicum*, *Colletotrictum gloeosporioides*, *Colletotrichum musae*	marine-sponge-derived	Hu et al. (2021)
*Phoma eupatorii* 8082		Antifungal: *Phytophthora infestans*		De Vries et al. (2018)
*Phoma multirostrata* XJ-2–1	Ergocytochalasin A	Antiviral: Human dengue virus type 3 (DV3), influenza A virus (H1N1), respiratory syncytial virus (RSV)		Peng et al. (2020)
*Phoma* sp.	Phomalacton, (3R)-5-hydroxymellein, emodin	Antibacterial: *Microbotryum violaceum, Bacillus megaterium*	*Fucus serratus*	Hussain et al. (2014)
*Phoma* sp. WF4	Viridicatol, tenuazonic acid, alternariol, alternariol monomethyl ether (	Antifungal: *F. graminearum*	*Eleusine coracana*	Mousa et al. (2015)
*Phoma* sp.	Phomapyrrolidones A, B and C	Antibacterial: *Mycobacterium tuberculosis*	*Saurauia scaberrinae*	Wijeratne et al. (2013)
*Phoma sp.*	4-hydroxymellein	Antibacterial: *B. subtilis*	*Cinnamomum mollissimum*	Santiago et al. (2014)
	4,8-dihydroxy-6-methoxy-3-methyl-3,4-dihydro-1H-isochromen-1-one	Antifungal: *Aspergillus niger*		
*Phoma* sp.	Thiodiketopiperazine deriva- tives	Antibacterial: *S. aureus, Streptococcus* *pyogenes*	*Glycyrrhiza glabra*	Arora et al. (2016)
*Phoma* sp.	phomafungin	Antifungal: *Candida albicans, Aspergillus fumigatus, Trichophyton mentagrophytes*	Africa and the Indian and Pacific Ocean islands	Herath et al. (2009)

Several members of the genus *Phoma* are well-known to produce a wide range of antimicrobials that are specific to the target organisms (bacteria, fungi, and viruses). *P. exigua* var. *exigua* produces antibiotic E and cytochalasin B (Boerema and Howeler 1967), *P. pigmentivora* produces LL-D253alpha (McIntyre et al. 1984), *P. lingam* (Tode) Desm. yields phomenoic acid and phomenolactone which are antibacterial and antifungal compounds (Topgi et al. 1987). In addition, there are other bioactive compounds reported from *Phoma* spp. For example, a well-known anti-infective agent squalestatin was reported from a *Phoma* spp. (Dawson et al. 1992); antitumor compound fusidienol A from another *Phoma* spp. (Singh et al. 1997), and Yamaguchi et al. (2002) isolated the bioactive compound FOM-8108 which inhibited neutral sphingomyelinases.

## Biosynthesis of silver nanoparticles by *Phoma* spp. and its antimicrobial efficacy

**Fig. 6:**
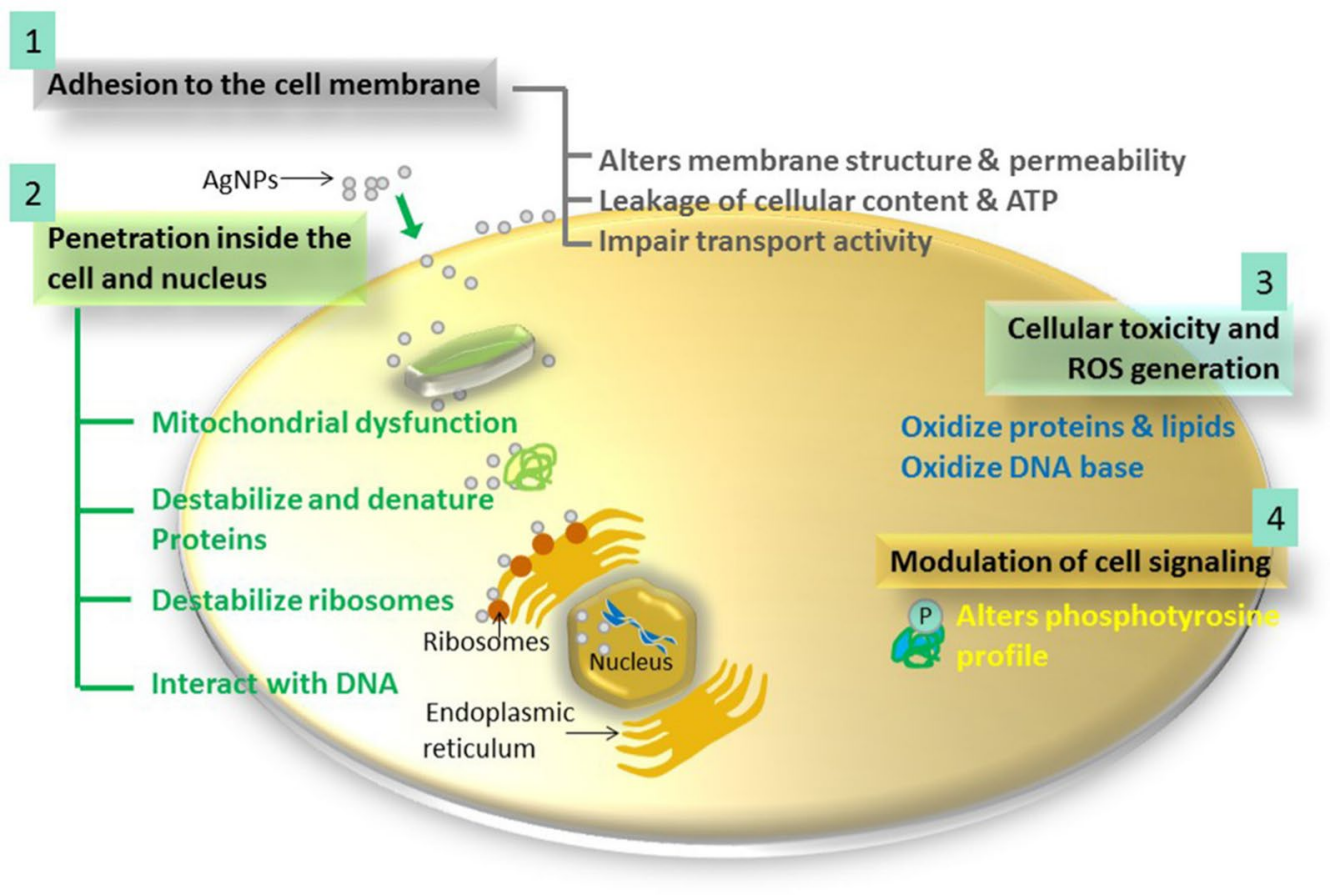
The four most prominent routes of antimicrobial action of AgNPs. 1. AgNPs adhere to microbial cell surface and results in membrane damage and altered transport activity; 2. AgNPs penetrate inside the microbial cells and interact with cellular organelles and biomolecules, and thereby, affect respective cellular machinery; 3. AgNPs cause increase in ROS inside the microbial cells leading to cell damage and; 4. AgNPs modulate cellular signal system ultimately causing cell death. Reproduced from Dakal et al. (2016) under the Creative Commons Attribution Licence (CC BY)

As discussed earlier, *Phoma* species are known to produce a wide range of metabolites that have already shown antimicrobial activity (Rai et al. 2009a). Some of the metabolites may not directly reveal the antimicrobial potential but can be used for the fabrication of silver nanoparticles (AgNPs) which also demonstrated remarkable antimicrobial potential. AgNPs are well known as a new generation of antimicrobials (Rai et al., 2009b). An elaborative account of multiple modes of action of AgNPs is reviewed by Dakal et al. (2016) and a schematic representation of the same is given in Fig. 6.

*Phoma* species are capable of extracellular synthesis of spherical AgNPs and silver nanorods. Extracellular synthesis of nanoparticles by *Phoma* spp. offer an advantage of obtaining large quantities of AgNPs at a rapid rate and in a relatively pure state. Furthermore, the extracellular synthesis of AgNPs by *Phoma* spp. would make the process simple and easier for downstream processing; fungal broths can be easily filtered by filter press of similar simple equipment, thus making it a cost-effective process (Gade et al., 2010). Moreover, the fabrication of AgNPs by *Phoma* spp. is a green and eco-friendly approach as no toxic chemicals, high temperature, or pressure are used for the synthesis (Gade et al., 2014; Rai et al. 2021a, b).

In a study, the fabrication of AgNPs by *P. glomerata* (MTCC-2210) was reported by Birla et al. (2009). Authors also reported the combined activity of commercial antibiotics and AgNPs synthesised from *Phoma* spp. by testing against *E. coli* JM-103 (ATCC-39403) and *S. aureus* (ATCC-25923) on Muller–Hinton agar plates. Commercial antibiotics like ampicillin (10 µg), gentamycin (10 µg), kanamycin (30 µg), streptomycin (10 µg) and vancomycin (30 µg) were used in the study. The comprehensive fold increases in area were observed for ampicillin, streptomycin, and vancomycin. Thus, the combined activity observed was better in *E. coli* than *S. aureus*. Whereas the disc diffusion analysis of only AgNPs showed better activity against *S. aureus* as compared to *E. coli*. In another study, the AgNPs synthesised from *P. gardinae* (ITCC 4554) showed antimicrobial activity against human pathogenic bacteria and fungi (Rai et al., 2015a). Authors evaluated the activity of AgNPs against *C. albicans*, *S. choleraesuis*, *P. aeruginosa*, *S. aureus,* and *E. coli*. The AgNPs were found to be most effective against *E. coli* followed by *S. aureus*, *C. albicans*, *S. choleraesuis,* and *P. aeruginosa* as compared with antibiotics. Further extracellular synthesis of AgNPs by *P. capsulatum*, *P. putaminum*, and *P. citri* was reported by Rai and co-workers (2015b). The AgNPs syjthesised from these *Phoma* spp. showed potential antimicrobial activity against *Aspergillus niger*, *C. albicans*, *S. choleraesuis*, *P. aeruginosa*, *S. aureus,* and *E. coli*. The least minimal inhibitory concentration (MIC) of 0.85 μg/ml was shown by AgNPs synthesized from *P. citri* against *S. choleraesuis*. AgNPs fabricated using *Phoma* spp. is not only reported for antibacterial and antifungal activity but also demonstrated antiviral potential. Some *Phoma* spp. isolated from the infected plants and identified on the basis of morphological and molecular characteristics were used for the fabrication of AgNPs. This demonstrated a significant decrease in replication efficiency for Herpes Simplex Virus (HSV)-1 and human parainfluenza virus (HPIV) type-3, and a minor effect on the replication of HSV-2 at a concentration of 10 mg/ml (Gaikwad et al. 2013). Further, the authors reported that AgNPs ability to control viral infectivity was most likely attributed to the size and zeta potential of the fabricated AgNPs, which interfere with virus and cell interaction, thereby blocking viral entry into the cell.

**Fig. 7:**
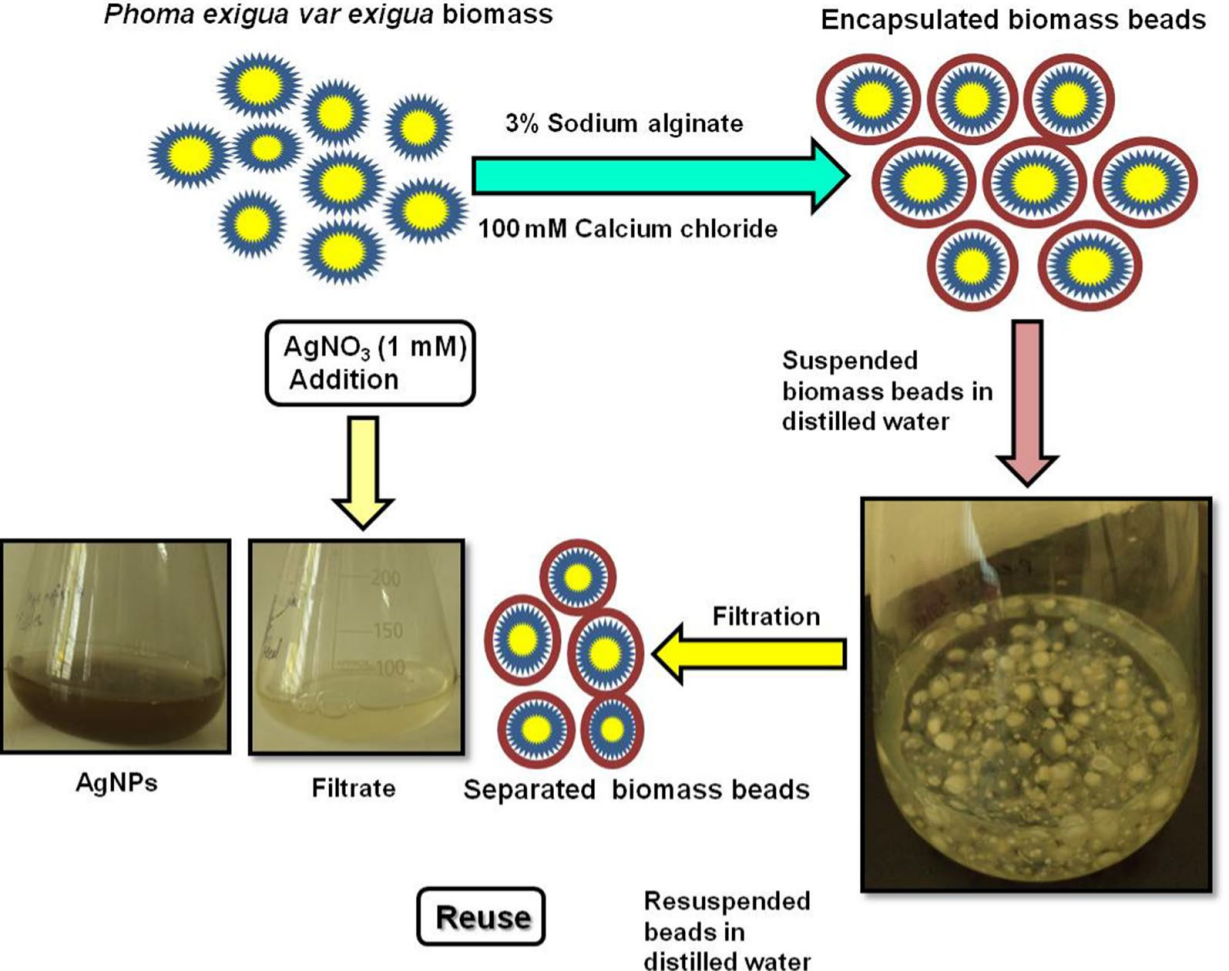
Graphical illustration of *Phoma exigua* var. *exigua* biomass immobilization process and AgNPs fabrication (Shende et al. 2017), reprinted with permission

Shende et al. (2017) synthesised AgNPs using immobilized biomass of the *P. exigua* var. *exigua.* This process was found to be a simple, fast, large-scale, and efficient route for the synthesis of AgNPs, without disintegration of calcium alginate beads in the medium for ten batch cycles. The immobilization of *P. exigua* biomass leads to the development of a method for the continuous synthesis of AgNPs. Moreover, this large-scale synthesis process could be a boon to the commercial fabrication of AgNPs which will be required due to the application of AgNPs in a large number of commercial products. The AgNPs thus produced also demonstrated antibacterial activity against *E. coli* and *S. aureus.* Graphical illustration of *P. exigua* var. *exigua* biomass immobilization process and AgNPs fabrication is given in Fig. 7.

It is evident from the above reports that the different *Phoma* spp. possess tremendous ability to reduce the inorganic metal ions to nanoparticles in general and AgNPs in particular, which is due to the extracellular secretions of metabolites by *Phoma* spp. Consequently, the metabolites secreted by the *Phoma* spp. can be harnessed and explored for the synthesis of nanoparticles of different sizes and shapes. In near future, the possibility of utilizing antimicrobial metabolites secreted by *Phoma* spp. for the fabrication of AgNPs cannot be overlooked, since these metabolites can be used with AgNPs synergistically which will provide the solution to the increasing drug resistance problem worldwide.

## Conclusions

Antimicrobial resistance and the entry of new fatal microbes like Coronavirus have made the researchers to seriously think about searching for new strategies to combat the global problem. Thus, there is a high demand for new antibiotics for difficult-to-treat bacteria and other pathogenic microbes. In this context, various fungi including *Phoma* offers antimicrobial metabolites. Various  species of *Phoma* particularly pigment-producing species such as *P. arachidicola, P. sorghina, P. exigua* var. *exigua*, *P. herbarum, P. multirostrata, P. betae,* and *P. fimeti* have already demonstrated their potential against pathogenic fungi, bacteria, and viruses. Moreover, several species of *Phoma* have been studied for the production of bioactive compounds such as polyketides, ergocytochalasin A, macrosporin, thiodiketopiperazines, terpenes, terpenoids, and alkaloids which have shown their antimicrobial potential. These antimicrobial metabolites of *Phoma* spp. are not only terrestrial but also include marine and endophytic spp. dwelling in medicinal plants. Moreover, some *Phoma* species are also known to synthesize silver nanoparticles extracellularly which have already proven to be the new generation of antimicrobials. Such a process of nanoparticle synthesis is eco-friendly, economically viable and a greener approach without the use of harmful chemicals and high pressure and temperature. These nanoparticles can also be utilized as nanocarriers for the slow and sustained delivery of antimicrobial drugs. Finally, more thorough research is required to screen different species of *Phoma* from extreme environments to find out potential antibiotic producers.
